# Antimicrobial Activity of *Artemisia dracunculus* Oil-Loaded Agarose/Poly(Vinyl Alcohol) Hydrogel for Bio-Applications

**DOI:** 10.3390/gels10010026

**Published:** 2023-12-28

**Authors:** Cristina Mihaela Rîmbu, Diana Serbezeanu, Tăchiță Vlad-Bubulac, Dana Mihaela Suflet, Iuliana Motrescu, Constantin Lungoci, Teodor Robu, Narcisa Vrînceanu, Mariana Grecu, Andreea Paula Cozma, Lenuța Fotea, Dragoș Constantin Anița, Ivona Popovici, Cristina Elena Horhogea

**Affiliations:** 1 Department of Public Health, Iasi “Ion Ionescu de la Brad” University of Life Sciences, 8 Sadoveanu Alley, 707027 Iasi, Romania; chorhogea@uaiasi.ro; 2 Department of Polycondensation and Thermally Stable Polymers, “Petru Poni” Institute of Macromolecular Chemistry, Grigore Ghica Voda Alley 41A, 700487 Iasi, Romania; tvladb@icmpp.ro (T.V.-B.); dsuflet@icmpp.ro (D.M.S.); 3 Department of Exact Sciences, Iasi “Ion Ionescu de la Brad” University of Life Sciences, 3 Sadoveanu Alley, 700490 Iasi, Romania; imotrescu@uaiasi.ro (I.M.); a.cozma@uaiasi.ro (A.P.C.); 4 Department of Plant Science, Iasi “Ion Ionescu de la Brad” University of Life Sciences, 3 Sadoveanu Alley, 700490 Iasi, Romania; constantinlungoci@uaiasi.ro (C.L.); teorobu@uaiasi.ro (T.R.); 5 Department of Industrial Machines and Equipments, Faculty of Engineering, “Lucian Blaga” University of Sibiu, 10 Victoriei Blvd, 550024 Sibiu, Romania; vrinceanu.narcisai@ulbsibiu.ro; 6 Department of Pharmacology, Iasi “Ion Ionescu de la Brad” University of Life Sciences, 8 Sadoveanu Alley, 707027 Iasi, Romania; mgrecu@uaiasi.ro; 7 Department of Animal Resources and Technologies, “Ion Ionescu de la Brad” University of Life Sciences, 700490 Iasi, Romania; fotealenuta@yahoo.com; 8 Regional Center of Advanced Research for Emerging Diseases Zoonoses and Food Safety (ROVETEMERG), “Ion Ionescu de la Brad” University of Life Sciences, 3 Mihail Sadoveanu Alley, 700490 Iasi, Romania; danita@uaiasi.ro; 9 Department of Preclinics, Iasi “Ion Ionescu de la Brad” University of Life Sciences, 8 Sadoveanu Alley, 707027 Iasi, Romania; ivona.popovici@uaiasi.ro

**Keywords:** *Artemisia dracunculus* essential oil, phytochemicals, hydrogels, antimicrobial activity

## Abstract

In this study, the potential use of *Artemisia dracunculus* essential oil in bio-applications was investigated. Firstly, the phytochemicals from *Artemisia dracunculus* were analyzed by different methods. Secondly, the *Artemisia dracunculus* essential oil was incorporated into the hydrogel matrix based on poly(vinyl alcohol) (PVA) and agar (A). The structural, morphological, and physical properties of the hydrogel matrix loaded with different amounts of *Artemisia dracunculus* essential oil were thoroughly investigated. FTIR analysis revealed the successful loading of the essential oil *Artemisia dracunculus* into the PVA/A hydrogel matrix. The influence of the mechanical properties and antimicrobial activity of the PVA/A hydrogel matrix loaded with different amounts of *Artemisia dracunculus* was also assessed. The antimicrobial activity of *Artemisia dracunculus* (EO *Artemisia dracunculus*) essential oil was tested using the disk diffusion method and the time-kill assay method after entrapment in the PVA/A hydrogel matrices. The results showed that PVA/agar-based hydrogels loaded with EO *Artemisia dracunculus* exhibited significant antimicrobial activity (log reduction ratio in the range of 85.5111–100%) against nine pathogenic isolates, both Gram-positive (*S. aureus*, *MRSA*, *E. faecalis*, *L. monocytogenes*) and Gram-negative (*E. coli*, *K. pneumoniae*, *S. enteritidis*, *S. typhimurium*, and *A. salmonicida*). The resulted biocompatible polymers proved to have enhanced properties when functionalized with the essential oil of *Artemisia dracunculus*, offering opportunities and possibilities for novel applications.

## 1. Introduction

*Artemisia dracunculus* is a species of the *Asteraceae* family with already proven numerous biological activities. It is a common plant with a wide geographical spread in Europe and America [1], North Africa, and Australia [2], and is extremely popular in Asian countries such as India [3]. Tarragon grows in any climatic and biogeographical area, with optimal development in any type of soil, although it prefers alkaline ones, having increased tolerance to extreme temperature and light conditions [4,5]. *Artemisia dracunculus*, along with other *Artemisia* species, has tremendous medical potential, as well as in the cosmetics or food industry [2]. Due to its volatile components, tarragon has long been treated as a spice, being recognized and used for its flavoring properties in the food industry [3,6].

This plant has equally impressive therapeutic properties, being an alternative in traditional medicine, especially Asian culture, used as a remedy in inflammatory processes, febrile states, digestive disorders, and parasitosis, its efficiency is well recognized for anesthetic [7,8], hypnotic, or antiepileptic effects [3]. In the Iranian culinary tradition, the consumption of dried or fresh tarragon leaves is recommended for their proven anticoagulant properties and significant improvement of HDL-cholesterol [9], which results in astatistically low rate of heart disease and atherosclerosis in the local population [10]. Similar scientific studies have confirmed these ethnopharmacological properties [5,11] and complemented the phytopharmaceutical activities of tarragon with its antidiabetic [12] and antioxidant potential [13,14,15], carminative or hepatoprotective capacity [6,15], and soothing and healing effects in allergic dermatitis [16] or dental diseases [5,6].

Relatively recent studies have highlighted the immunomodulatory effect of the plant [3,17] and the thyroid-stimulating potential, with applicability in the management of hypothyroidism [18]. In vitro and in vivo test models of plant extracts revealed the antitumor capacity of *Artemisia dracunculus* metabolites [19,20,21,22]. Research has also shown that artemisinin, a phytochemical compound found in many species of *Artemisia*, including *Artemisia dracunculus* [23], was able to induce a very good larvicidal effect towards the main vector (*Anopheles stephensi*) for malaria transmission to humans. It is known that artemisinin is an antiparasitic drug with excellent activity against cellular parasites, including the protozoan *Plasmodium falciparum*, the etiological agent of malaria [24]. Pharmacological studies on *Artemisia dracunculus* have shown that this plant is a complex source of active compounds with antimicrobial activity [5,14,25,26]. 

Hydrogels are polymeric, biocompatible, non-toxic, non-allergic networks, which have aroused a special interest through their versatile behavior, having various functional properties of absorption, porosity, and flexibility, and, through various physical and chemical interactions, have been used in various medical, agricultural, and biotechnological applications [27,28,29,30].

Encapsulation or conjugation of phytocompounds can optimize their physical and chemical properties, resulting in improved drug release and increased therapeutic outcomes. Many hydrogel systems have been obtained by incorporating phytocompounds into polymers [28].

Polyvinyl alcohol (PVA), a synthetic, semicrystalline, tasteless, odorless, water-soluble polymer structured with a repeated number of functional hydroxyl groups, was obtained for the first time by W.O. Herrmann et al. in 1924 using the polymerization of vinyl acetate (VAc) [31]. PVA-based hydrogels are excellent biomaterials due to their superior qualities, such as biocompatibility, absence of cytotoxicity [32,33], rapid biodegradability, insolubility in organic solvents, and mechanical strength [34]. Also, compared to other polymers, PVA-based hydrogels function as barriers against gases and show increased resistance to oils, fats, and solvents [35]. 

Agar is a heterogeneous phyto-polysaccharide obtained from red algae of the family *Rhodophyta*, containing two fractions: agarose and agaropectin [36]. Agarose is the major component of agar, identified as a neutral polymer structured by linear and repeating units made up of agarobiose disaccharide of a 3-O-linked β-D-galactopyranose residue, alternating with a 4-O-linked 3,6 anhydro-α-L-galactopyranose in a linear sequence [37]. Agaropectin, the second compound of agar, is an anionic polysaccharide, having the same backbone as agarose but containing acid groups such as sulphate, pyruvate, and glycuronate. Among these two components, agaropectin is a non-gelling compound responsible for the viscosity of agar, while agarose confers its gelling properties [36,38,39].

In addition to its extraordinary jellifying property, agar manifests a high thermal hysteresis (jellifies and melts at different temperatures) and complete reversibility of the gel [40], which makes it an excellent medium for electrophoresis [38]. It is also a common culture medium used to isolate bacteria, yeasts, or fungi. Due to its excellent properties, it has been successfully used as a stabilizer for emulsions and suspensions, a jellifying agent in the food industry, for encapsulating bioactive molecules in the pharmaceutical industry, as well as for many other applications in biotechnologies, tissue engineering [41,42], the medical field, cosmetics, and biofuel production [43].

Most often, agar hydrogels have been optimized by integrating other polymers to obtain an interpenetrating network, which facilitates the embedding and delivery of active biomolecules. The increased porosity of some agar hydrogels allows adhesion and cell multiplication or division [42].

Agar hydrogel has also been studied as a food packaging material, but in this regard, its thermolabile and brittle properties have been a disadvantage [39]. For this reason, agar hydrogel can be streamlined with other versatile polymers, such as PVA, to obtain new functional structures. Studies on combining and characterizing functionalized PVA/A hydrogels with phytoextracts are not very numerous [44,45,46]. 

This study aimed to develop a PVA/A hydrogel matrix with incorporated essential oil, aiming to use this new and improved system in the production of medical devices or as a biodegradable alternative to traditional packaging.

## 2. Results and Discussion

### 2.1. Preparation, Synthesis, and Structural Characterization of Artemisia dracunculus Essential Oil

**Figure 1:**
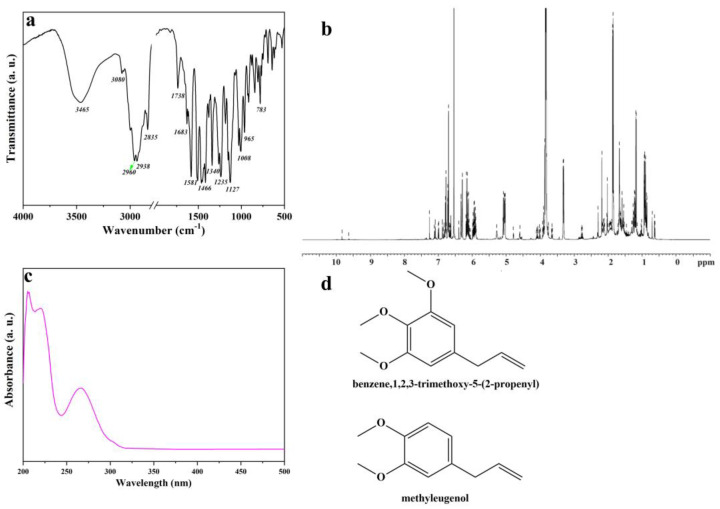
FTIR spectrum of *Artemisia dracunculus* essential oil (**a**), ^1^H NMR spectrum of *Artemisia dracunculus* essential oil (**b**), UV-VIS spectrum of *Artemisia dracunculus* essential oil (**c**), and chemical structure of the most important compounds of *Artemisia dracunculus* essential oil (**d**).

The *Artemisia dracunculus* essential oil was analyzed by Fourier transform infrared (FTIR) and ^1^H nuclear magnetic resonance (NMR) spectroscopy, UV-VIS spectrophotometry, and gas chromatography–mass spectrometry (GC-MS), aiming to identify the main chemical constituents of the *Artemisia dracunculus* essential oil. The FTIR analysis of *Artemisia dracunculus* essential oil confirmed the presence of alkenes, alcohols, ethers, carboxylic acids, esters, alkanes, and phenols, which show major peaks at 783, 965, 1008, 1127, 1235, 1340, 1466, 1581, 1683, 1738, 2835, 2938, 2960, 3090, and 3465 (Figure 1a). The broad absorption peak of nearly 3400 cm^−1^ corresponds to the—OH stretching vibrations. The presence of polyphenolic structures in the *Artemisia dracunculus* essential oil can also be observed in the FTIR spectra by the presence of absorption bands around 2900 cm^−1^ which are attributed to the asymmetric and symmetric stretching vibrations of the C–H groups. The presence of the C=C stretching vibrations [47] of the aromatic ring can be observed at 1581 and 1512 cm^−1^, respectively. In the research of Schulz, these bands were assigned to the aromatic ring of the polyphenolic compounds. Therefore, the FTIR analyses indicated the presence of benzene, 1,2,3-trimethoxy-5-(2-propenyl)/(E)-isoelemicin, and methyleugenol as the main chemical constituents of the *Artemisia dracunculus* essential oil.

As shown in Figure 1b, ^1^H NMR spectrum the protonic spectrum of *Artemisia dracunculus* essential oil presents a lot of overlapped peaks. However, it was possible to identify some compounds found in the *Artemisia dracunculus* essential oil. The UV-VIS spectrum (Figure 1c) of *Artemisia dracunculus* essential oil indicates peaks in the regions 200–210 nm, 230–235 nm (typical hydroxycinnamic acid), and 270–290 nm (flavanol derivatives). As presented in the literature, the peaks corresponding to flavone conjugates showed three absorptions at 210–230 nm, 250–280 nm, and 330–350 nm [48], similar to those found here. The spectra of the *Artemisia dracunculus* essential oil and standard compounds were compared, and the results led to the assignment of benzene,1,2,3-trimethoxy-5-(2-propenyl)/(E)-isoelemicin and methyleugenol (Figure 1d).

**Figure 2:**
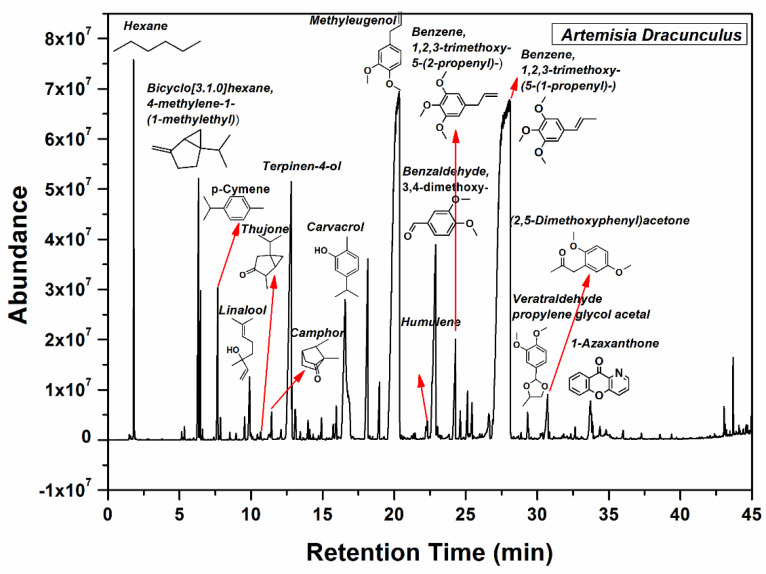
GC-MS spectrum of the *Artemisia dracunculus* essential oil.

The main components of the *Artemisia dracunculus* essential oil can be observed in Figure 2. The main botanical constituents of the essential oil were: bicyclo[3.1.0] hexane, 4-methylene-1-(1-methylethyl) (3.06%), *p*-cymene (1.24%), linalool (0.7%), camphor (0.33%), terpinene-4-ol (6.45%), phenol, 2-methyl-5-(1-methylethyl) (5.01%), 2,6-octadiene, 2,6-dimethyl (2.49%), geranyl acetate (0.51%), methyl eugenol (23.36%), benzene,1,2,3-trimethoxy-5-(2-propenyl)/elemicin (1.3%), (-)-Spathulenol (0.9%), benzalhyde,3,4,5-trimethoxy (0.78%), benzene,1,2,3-trimethoxy-5-(2-propenyl)/(E)-isoelemicin (38.89%), 2,5-dimethoxyphenyl)acetone (0.97%), 1-azaxanthone (0.89%), phthalic acid, di(2-propylpenthyl)ester. As can be seen in Figure 2, benzene,1,2,3-trimethoxy-5-(2-propenyl)/(E)-Isoelemicin, and methyl eugenol had the highest proportion in the oil, with other components being present in smaller amounts in the *Artemisia dracunculus* essential oil.

### 2.2. Preparation, Synthesis, and Structural Characterization of PVA/A Hydrogel Matrix and PVA/A Hydrogel Matrix Loaded with Artemisia dracunculus Essential Oil

**Figure 3:**
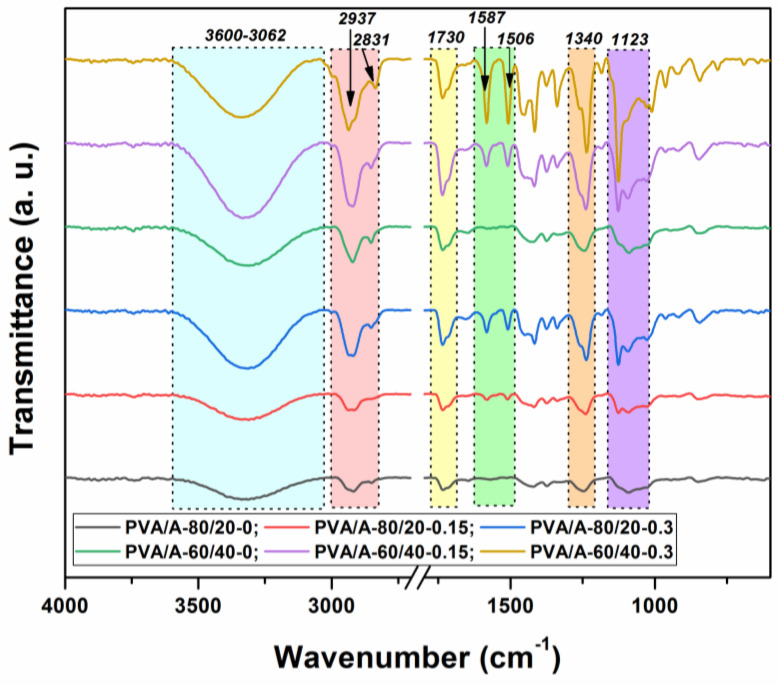
FTIR spectra of the PVA/A hydrogels and PVA/A hydrogels loaded with *Artemisia dracunculus essential oil*.

To determine the molecular interactions in the PVA/A hydrogel matrix and the PVA/A hydrogel matrix loaded with *Artemisia dracunculus* essential oil, an FTIR analysis was carried out. The FTIR spectra of all the samples investigated in this study are presented in Figure 3. In the FTIR spectrum of the PVA/A hydrogel matrix, characteristic PVA and A peaks were recorded as –OH bonds (3286 cm^−1^), C–H aliphatic asymmetric and symmetric stretching vibrations (2937 and 2831 cm^−1^), and C–O stretching vibrations (1123 cm^−1^). Meanwhile, the peak observed at 1340 cm^−1^ is indicative of bonding involving O–H and C–H.

In Figure 3, in the PVA/A hydrogel matrix loaded with *Artemisia dracunculus* essential oil, the presence of the two peaks at 1587 and 1506 cm^−1^ is due to stretching vibrations of C=C in the cyclic alkene. The stretching vibrations are characteristic of the carboxylic acid and aldehyde groups can be observed in the FTIR spectra at 1715 and 1730 cm^−1^. The presence of some new weak peaks in the range 615–950 cm^−1^ can be attributed to the C–H bonding vibrations, confirming once again the strong intermolecular interactions between *Artemisia dracunculus* essential oiland PVA/A hydrogel matrix.

**Figure 4:**
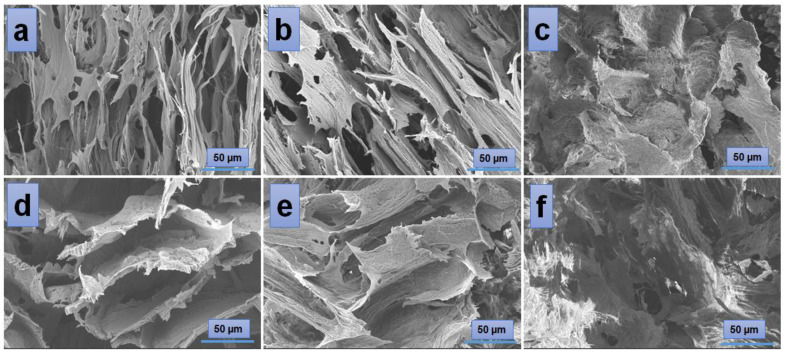
SEM images of the hydrogels: PVA/A-80/20-0 (**a**), PVA/A-60/40-0 (**d**), and hydrogels loaded with *Artemisia dracunculus essential oil*: PVA/A-80/20-0.15 (**b**), PVA/A-80/20-0.3 (**c**), PVA/A-60/40-0.15 (**e**), and PVA/A-60/40-0.3 (**f**).

**Figure 5:**
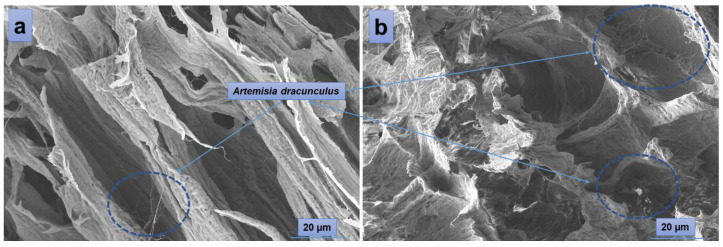
SEM images of PVA/A-80/20-0.15 (**a**) and PVA/A-80/20-0.3 (**b**).

According to the SEM images (Figure 4), the hydrogels have a porous morphology with interconnected pores and a rather uneven structure. All investigated samples showed contracted areas. These are the results of the physical cross-linking of PVA induced by the freeze-thaw technique used in the preparation of the PVA/A hydrogel matrix. SEM microimaging also shows that the incorporation of *Artemisia dracunculus* essential oilinto the PVA/A hydrogel matrix leads to the formation of porous, interlinked channels. Moreover, the presence of the EO *Artemisia dracunculus* is highlighted by its deposition in the form of particles or fibers inside the pores (Figure 5).

**Figure 6:**
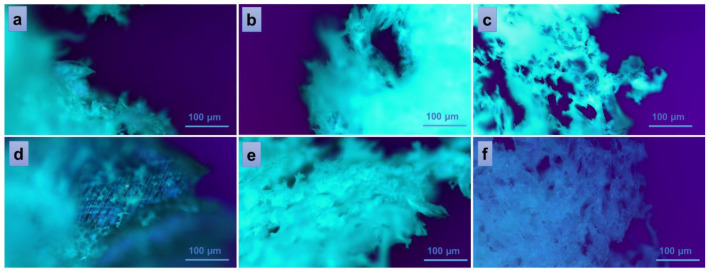
Polarized optical microscopy images of the hydrogels: PVA/A-80/20-0 (**a**), PVA/A-60/40-0 (**d**), and hydrogels loaded with *Artemisia dracunculus:* PVA/A-80/20-0.15 (**b**), PVA/A-80/20-0.3 (**c**), PVA/A-60/40-0.15 (**e**), and PVA/A-60/40-0.3 (**f**).

To further study some inner physical characteristics of the materials, such as surface roughness, channel sizes and shapes, porosity, and other factors that can affect material properties such as strength, stiffness, and permeability, polarized light microscopy was used. The technique is often useful to identify and characterize different types of materials based on their birefringence. The images presented in Figure 6 were collected at room temperature from every sample in the series PVA/A-80/20 (Figure 6a–c) and PVA/A-60/40 (Figure 6d–f), respectively. The white, fine wool-like material exhibited a fibrous appearance in the vicinity of fracture zones (Figure 6a,b,e). No significant differences were observed in the bulk morphologies of the samples PVA/A-80/20-0 and PVA/A-80/20-0.15, which contained a smaller amount of *Artemisia dracunculus* essential oil (Figure 6a,b). While continuing to add this compound to the PVA/A polymeric matrix, larger pores, and open channels could be observed in the sample PVA/A-80/20-0.30 (Figure 6c),these results being consistent with the SEM observations. An interesting behavior was observed in the case of the samples containing an increased ratio of agar, with this component being responsible for the augmented birefringence in the samples of the series PVA/A-60/40. Thus, some thin-layer areas of the introspected material revealed a more organized network-like morphology (Figure 6d). This behavior was diminished when the EO *Artemisia dracunculus* was introduced in the matrix, but still, the overall birefringence was superior in the series PVA/A-60/40 (Figure 6e,f) over the series PVA/A-80/20, suggesting some crystallinity of the materials containing a higher quantity of agar. Similar behavior was observed for sample PVA/A-60/40-0.30, containing a higher amount of EO *Artemisia dracunculus*, with the sponge-like appearance of the material revealing larger, translucent, and interconnected pores and channels (Figure 6f).

**Figure 7:**
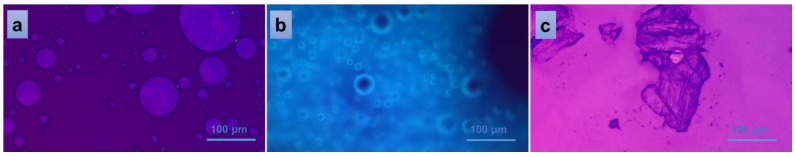
Polarized optical microscopy images of the interaction of PVA/A-80/20-0.15 hydrogel with water: isotropic liquid (**a**), uniform distribution of the EO (**b**), and solid, crystalline material resulted after the evaporation of the solvent (**c**).

Figure 7 indicates the behavior of PVA/A-80/20-0.15 material when interacting with distilled water. The addition of one drop of water on top of the fractured piece of material gravimetrically collapsed the material on the glass plate, revealing a homogeneous distribution of EO *Artemisia dracunculus* into the overall mass of the PVA/A-80/20 polymeric matrix (spherical EO bubbles in Figure 7b). When a second glass plate was placed above the system, an isotropic liquid populated the entire microscopic view, suggesting the homogeneity of the bulk material once again (Figure 7a). Finally, when detaching the second glass plate and giving the material time to dry in ambient conditions, a crystalline solid was obtained, confirming the non-semi-interpenetrating interaction between the components of the studied materials.

### 2.3. Mechanical Behavior

**Figure 8:**
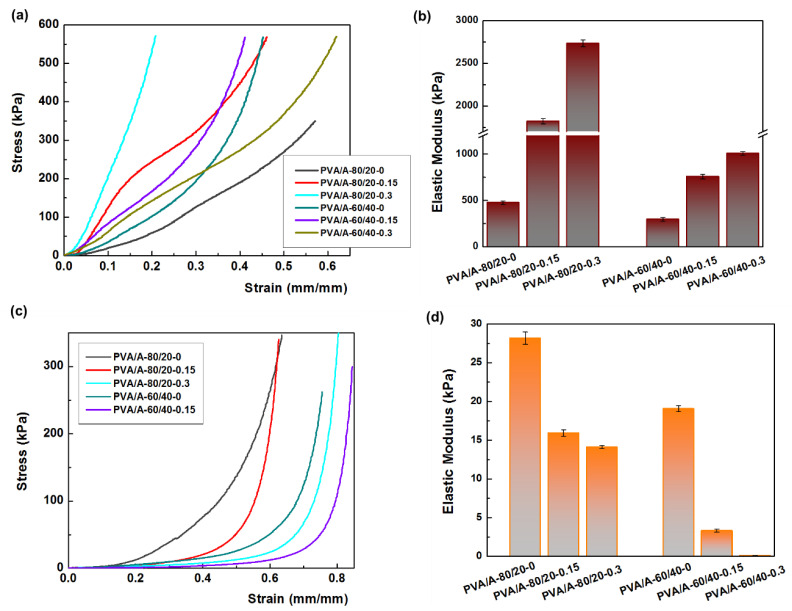
Mechanical properties of hydrogels. (**a**) the compressive stress-strain curves of dehydrate hydrogels; (**b**) the elastic modulus for dehydrated hydrogels; (**c**) the compressive stress-strain curves of hydrate hydrogels; (**d**) the elastic modulus for hydrated hydrogels.

Mechanical strength is a crucial property for the potential applications of PVA/A hydrogels. Uniaxial compressive tests were conducted on both dehydrated and hydrated samples, as shown in Figure 8. The hydration of PVA/A hydrogels occurred under controlled conditions at 75% relative humidity for 24 h at 25 °C. Figure 8a illustrates the compressive stress-strain curves of dehydrated PVA/A hydrogels. The elastic modulus values varied widely, ranging from 477 kPa for PVA/A-80/20-0 to 2535 kPa for PVA/A-80/20-0.3. In the case of hydrogels without *Artemisia dracunculus* essential oil, the elastic modulus decreased from 477 kPa for PVA/A-80/20-0 to 294 kPa for PVA/A-60/40-0, this being attributed to the hindrance of the agar of crystalline domain formation between the PVA chains during the freeze-thaw process [35,49], also confirmed by SEM microscopy (Figure 4a,d). The elastic modulus values for PVA/A hydrogels without essential oil aligned with literature data [48,50]. The addition of *Artemisia dracunculus* essential oil led to a substantial increase in the elastic modulus values to 1823 kPa and 2734 kPa for PVA/A-80/20-0.15 and PVA/A-80/20-0.3, respectively, in the case of dehydrated hydrogels (Figure 8b). This behavior could be attributed to intermolecular hydrophobic interactions between hydrophilic polymer chains (PVA and agar) and hydrophobic compounds from *Artemisia dracunculus* essential oil. The compressive stress-strain curves of the hydrated PVA/A hydrogels showed a noticeable reduction in the modulus of elasticity to 28.16–14.11 kPa and 19.04–3.33 kPa for PVA/A-80/20 and PVA/A-60/40, respectively (Figure 8c). This observation is further emphasized in Figure 8d. The hydrogels became soft, and during the compression test, the samples showed no cracks. Notably, the compression test could not be performed for PVA/A-60/40-0.3 hydrogel due to its highly soft nature.

### 2.4. In Vitro Evaluation of the Antimicrobial Potential of Essential Oil Artemisia dracunculus Essential Oil

Testing the antimicrobial susceptibility of *Artemisia dracunculus* essential oil using the diffusion technique revealed its antimicrobial potential, which was quantified by measuring the diameter of the bacterial inhibition zones and calculating the mean and standard error of the values obtained in triplicate tests (Table 1).

**Figure 9:**
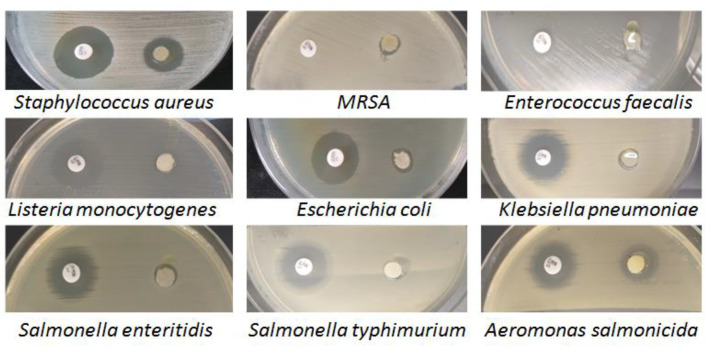
The microbial activity of *Artemisia dracunculus* essential oil (10 µL) and the positive control (gentamicin, 10 µg) was evaluated by the disc diffusion method.

The positive control for antimicrobial efficacy testing was gentamicin (10 µg), an aminoglycoside antibiotic recommended by the international standards for antimicrobial susceptibility testing [51] and used in anti-infective therapy in animals and humans [52]. All pathogenic bacterial isolates were sensitive to the action of *Artemisia dracunculus* essential oil, with average areas of inhibition ranging from 7.3–13.3 mm for Gram-positive species to 7.2–16.9 mm for Gram-negative species (Figure 9).

The best antimicrobial activity was identified in *Aeromonas salmonicida* (16.9 mm) and *MRSA* (13.3 mm). The variability of the results shows that the effect of *Artemisia dracunculus* EO (10 µL) was influenced by the tested bacterial species and not by the bacterial group to which it belonged, although the lowest susceptibility was found in two Gram-negative species: *Salmonella enteritidis* (7.4 mm) and *Klebsiella pneumoniae* (7.2 mm) and only one Gram-positive species: *Listeria monocytogenes* (7.4 mm). It is known that Gram-negative bacteria are generally more resistant to the effects of the antimicrobial phytocompounds in essential oils than Gram-positive bacteria due to the different structure of the cell wall [53].

### 2.5. In Vitro Evaluation of the Antimicrobial Potential of PVA/A Hydrogel and PVA/A Loaded with Artemisia dracunculus Essential Oil Hydrogel

Different amounts of *Artemisia dracunculus* EO (1.5 and 3 mL) embedded in two different hydrogel matrices of PVA/A were tested against the nine clinical isolates also used for the diffusion tests of *Artemisia dracunculus* EO (see previous method). The quantitative technique in liquid medium (time kill assay) provided us with high-precision data on the antimicrobial potential of the tested hydrogel matrices: PVA/A-80/20-0.15, PVA/A-80/20-0.3, PVA/A-60/40-0.15, and PVA/A-60/40-0.3, evaluated after 6, 12, 24, and 48 h of contact with the microbial suspension. The positive control was represented by the density of the bacterial suspension (1.5 × 10^8^ CFU/mL).

All numerical results were converted to logarithmic values, for which the logarithmic reduction (%Lg.red) and the logarithmic reduction percentage (%Lg.red) were calculated (Table S1).

The overall analysis of the data shows us that the values obtained as a result of antimicrobial inhibition are not always correlated with the amount of *Artemisia dracunculus* EO. Table S1 shows the results obtained after 24 (T3)–48 (T4) hours of contact, since after 6 (T1) and 12 h (T2), the degree of microbial inhibition was not quantifiable for most of the bacterial species tested (Figure S1).

All types of PVA/A loaded with *Artemisia dracunculus* EO hydrogels produced a logarithmic reduction between 90–99.9999%, except for PVA/A-80/20-0.15 (85.5111%) and PVA/A-60/40-0.15 (86.5777%) against *Klebsiella pneumoniae* and PVA/A-80/20-0.15 (86.0666%) against *Salmonella enteritidis* after 24 h of contact. These results demonstrate the bacteriostatic capacity of *Artemisia dracunculus* essential oil.

The bactericidal activity, which consisted of a logarithmic reduction of 100%, was determined sporadically for *Listeria monocytogenes* after 24 h of contact with PVA/A-80/20-0.3 and after 48 h with PVA/A-80/20-0.3 and PVA/A-60/40-0.15, respectively, for *Enterococcus faecalis* after 48 h of contact with PVA/A-80/20-0.3, and for methicillin-resistant *Staphylococcus aureus* (MRSA) after 48 h of contact with PVA/A-60/40-0.30 hydrogel.

A remarkable result was obtained for all types of PVA/A loaded with *Artemisia dracunculus* essential oil hydrogels that produced complete microbial inhibition only after 6 h of contact with the bacterial suspension of *Aeromonas salmonicida* (Figure S1).

The hydrogel matrices PVA/A-80/20-0 and PVA/A-60/40-0 (the control hydrogel) showed no antimicrobial activity, indicating that the inhibitory effect of the microbial culture was due to the essential oil of *Artemisia dracunculus* and not the hydrogels themselves. Figure S1 shows the microbial inhibition by all PVA/A and PVA/A+EOA.dr. hydrogel matrices evaluated by contact times T1 (6 h), T2 (12 h), T3 (24 h), and T4 (48 h).

The antimicrobial role of polyphenolic phytoconstituents, alkaloids, carotenoids, terpenes, or molecules with sulphur-containing groups has been recognized [54], but the mechanisms of antimicrobial action of EO *Artemisia dracunculus* have not been fully understood [55].

The antimicrobial efficacy of tarragon has been evaluated over time, but the results are heterogeneous due to the different test methods, the bacterial species studied, the extraction method, as well as the phytochemical composition of the plant, which in turn is influenced by the geographical origin [54,56,57].

The composition of agar optimized the properties of PVA, resulting in two hydrogel matrices (PVA/A-80/20 and PVA/A-60/40) with excellent physicochemical and mechanical properties. The advantages of combining these two biocompatible polymers have also been used in other experimental models, demonstrating the versatility of these types of hydrogels [44,58,59,60,61,62].

## 3. Conclusions

In conclusion, this study focused on *Artemisia dracunculus*, aiming to explore its potential applications in the biomedical field or food technology. The investigation involved the analysis of phytochemicals in *Artemisia dracunculus* using various methods. Subsequently, *Artemisia dracunculus* essential oil was successfully incorporated into hydrogel matrices based on poly(vinyl alcohol) (PVA) and agar (A). The comprehensive examination of the structural, morphological, and physical properties of the hydrogel matrices, loaded with varying amounts of *Artemisia dracunculus* essential oil, provided valuable insights. FTIR spectra confirmed the successful loading of *Artemisia dracunculus* essential oil into the PVA/A hydrogel matrices. This study also delved into the mechanical properties and antimicrobial activity of the PVA/A hydrogel matrices loaded with different amounts of *Artemisia dracunculus*. The antimicrobial efficacy of *Artemisia dracunculus* essential oil was evaluated using both the disk diffusion and time-kill assay methods after entrapment into PVA/A hydrogel matrices. The results demonstrated significant antimicrobial activity of PVA/agar-based hydrogels loaded with *Artemisia dracunculus* essential oil, showing a log reduction ratio in the range of 85.51–100% against various Gram-positive and Gram-negative pathogenic isolates. The outcomes suggest a favorable outlook for the synergistic integration of these biocompatible polymers, while the incorporation of *Artemisia dracunculus* essential oil introduces novel avenues for practical applications. The formulated design, resulting from the harmonious combination of these biocompatible polymers and the functionalization with *Artemisia dracunculus* essential oil, not only demonstrates promise but also establishes the groundwork for the development of innovative and effective systems with potential applications in the biomedical field or food technology.

## 4. Materials and Methods

### 4.1. Materials

The aerial parts (herbs) of the *Artemisia dracunculus* plants were obtained from the collection of medicinal and aromatic plants at the University of Life Sciences (IULS, Romania). A total of 1000 g of fresh plant material was used to extract the essential oil using hydro-distillation (3 h) in a large-capacity Clevenger-type apparatus. The oil was obtained and then stored at 4 °C in opaque vials.

PVA powder with different weight average molecular weights (M_w_ = 30,000–70,000 Da) and different degrees of hydrolysis (87–90%) was purchased from Sigma-Aldrich (Sigma-Aldrich Chemie GmbH, Eschenstraße 5, 82024 Taufkirchen, Germany). Purified agar (Oxoid Ltd., Hampshire, UK) (Molecular Weight: 336.33 g/mol) was purchased from Thermo Fisher Scientific (Basingstoke, UK).

### 4.2. Methods

#### 4.2.1. FTIR Investigation

FTIR spectra of electrospunfibres were obtained by using a BioRad ‘FTS 135’ FTIR spectrometer equipped with a Specac “Golden Gate” ATR accessory. A LUMOS Microscope Fourier Transform Infrared (FTIR) spectrophotometer (Bruker Optik GmbH, Ettlingen, Germany), equipped with an attenuated total reflection (ATR) device, was used to record the scans between 4000 and 500 cm^−1^ at a resolution of 4 cm^−1^.

#### 4.2.2. ^1^H NMR Investigation

The NMR spectra have been recorded on a Bruker Avance Neo instrument (Bruker BioSpin, Rheinstetten, Germany) operating at 400.1 MHz for ^1^H. The samples were solubilized in CDCl_3_ and transferred into 5 mm Wilmad 507 NMR tubes, then recorded with a 5 mm four-nuclei direct detection z-gradient probe for ^1^H. Chemical shifts reported in δ units (ppm) were referenced to the internal deuterated solvent CDCl_3_, referenced at 7.26 ppm for ^1^H NMR.

#### 4.2.3. UV-VIS Investigation

Ultraviolet-visible (UV-VIS) spectra of essential oil were recorded using a UV spectrophotometer (UV Jenway 7305). The interval for scans was in the spectral range of 200–500 nm. The test was repeated three times for each sample.

#### 4.2.4. GC-MS Analysis

The analysis of *Artemisia dracunculus* essential oil by gas chromatography was carried out using an Agilent 6890N Chromatograph equipped with a flame ionization detector (FID) and an Agilent 5975 inert XL mass detector (MSD) with an ionization energy of 70 eV. Two Teknokroma TR-520232 capillary columns were used. The analysis conditions were: an initial temperature of 40 °C for 14 min; a gradient of 1.5 °C/min up to 250 °C; and an isotherm for 10 min. The injector (and transfer line) temperature was 270 °C. Helium was used as a carrier gas for the 2 L of injected *Artemisia dracunculus* essential oil in splitless mode at a flow rate of 0.5 mL/min. GC-MS analysis was performed by ESI in a mass range of 24 to 400 *m*/*z* with a delay time of 2 min. Identification of *Artemisia dracunculus* components was performed by comparing the mass spectra to those in the available NIST database (GC-MS) and by their relative retention index (Kovacs Index, KI) towards a mixture of alkanes C_4_ to C_26_, injected in the same experimental conditions (GC-FID).

#### 4.2.5. Scanning Electron Microscopy Investigation

Microscopic investigations for all the studied samples presented in this paper and of their corresponding chars were performed on an Environmental Scanning Electron Microscope Type Quanta 200, operating at 10 kV with secondary electrons in low vacuum mode (LFD detector).

#### 4.2.6. Polarized Optical Microscopy

Optical microscopy microphotographs were obtained from all the studied samples at ambient temperature to further investigate their morphology using a Zeiss Microscope Axio Imager A2M fitted with aLinkam Plate LTS420 with a 10× objective (Carl Zeiss Microscopy GmbH, Oberkochen, Germany). Small pieces of the bulk dry hydrogel were pulled out using tweezers, placed on tiny glass plates, and examined with the microscopic view set at the edge of the torn material before the photos of each sample in the series were taken.

#### 4.2.7. Mechanical Properties of the PVA/A Hydrogels

The mechanical testing was performed using a Texture Analyser (Brookfield Texture PRO CT3^®^, Brookfield Engineering Laboratories Inc., Middleborough, MA, USA) at room temperature, following the ASTM D882 standard. The compression tests were performed with rectangular samples (10 × 10 mm^2^ and 5 mm depth) with a trigger load of 0.067 N and a compression rate of 0.2 mm/s. Mechanical tests were performed both with dry and hydrated samples under controlled conditions (75% relative humidity and 25 °C). Compression stress and strain values were calculated following the procedure already reported in the literature [35]. The elastic modulus (E, kPa) was calculated from the slope of the linear stress-strain curves between 2 and 8% compressions.

#### 4.2.8. Preparation of the Hydrogels

Two stock solutions were prepared, as follows: The first solution was made by dissolving 5.55 g of PVA in 50 mL of double-distilled water. For complete dissolution, the mixture was heated to 90 °C for 2 h. After heating, a clear, transparent solution resulted. The second solution was made by dissolving 0.51 g of agar in 25 mL of double-distilled water and heating the mixture at 60 °C for 2 h. The two stock solutions were used to prepare two new hydrogels. The first hydrogel was prepared using 1.4 mL of the first stock solution and 2.1 mL of the second one, then mixed using an ultrasonic bath for 10 min. The obtained mixture was transferred into a 10 mL Berzelius glass, and three freeze-thaw cycles were applied to this mixture. After the three cycles, the hydrogel (PVA/A-80/20-0) was dried by lyophilization. The obtained product was kept in a desiccator until analysis. The same procedure was performed for the second hydrogel (PVA/A-60/40-0), using this time 3 mL of stock solution 1 and 2 mL of stock solution 2 (Table 2).

#### 4.2.9. Preparation of the PVA/A Loaded with *Artemisia dracunculus* Essential Oil Hydrogels

Different amounts of *Artemisia dracunculus* essential oil were added to the PVA/A-80/20-0 and PVA/A-60/40-0 solutions, and the hydrogels loaded with *Artemisia dracunculus* were prepared in a similar way to the hydrogels presented above. Table 1 lists the amounts of *Artemisia dracunculus* incorporated in the two hydrogels.

#### 4.2.10. Antimicrobial Activity

##### Bacterial Strains

The antimicrobial potential of *Artemisia dracunculus* essential oil (EO *Artemisia dracunculus*) and PVA/A loaded with *Artemisia dracunculus* hydrogels was tested against four Gram-positive strains: *Staphylococcus aureus*, Methicillin-resistant *Staphylococcus aureus*, *Enterococcus faecalis*, and *Listeria monocyogenes*, and five Gram-negative strains: *Escherichia coli*, *Klebsiella pneumoniae*, *Salmonella enteritidis*, *Salmonella typhimurium*, and *Aeromonas salmonicida*. The nine tested strains were pathogenic clinical isolates obtained from various pathological materials (purulent exudate, sputum, pharyngeal exudate, urine, and coprocultures) from domestic animals. The bacterial species were identified using matrix-assisted laser desorption/ionisation time-of-flight mass spectrometry (MALDI-TOF MS, Bruker Biotyper^®^ Sirius, Bremen, Germany) at the Regional Centre of Advanced Research for Emerging Diseases, Zoonoses, and Food Safety (University of Life Sciences, Iasi, Romania).

##### Antimicrobial Susceptibility Testing Using the Diffusion Method

The Kirby–Bauer diffusion method is a standardized procedure for testing the susceptibility of microorganisms to antibiotics that provides reproducible and easy-to-interpret results. The working principle consists of applying the samples to be tested to the surface of a solid culture medium that has previously been cultivated with a microbial culture. Bacterial suspensions with a cell density corresponding to the turbidity of the 0.5 McFarland standard (1.5 × 10^8^ bacterial cells/mL) were prepared from 24 h cultures. An inoculum of the bacterial suspension was spread on the surface of the culture medium Mueller Hinton Agar (Oxoid, UK) and initially distributed in sterile Petri plates (90 mm). After drying, sterile filter paper discs (diameter 6 mm) resembling the antibiotic disc (Gentamicin, 10 µg, Oxoid, UK) were spread on the surface of the medium, to which *Artemisia dracunculus* EO (10 μL) was added. The antimicrobial effect of *Artemisia dracunculus* EO was compared with that of gentamicin (positive control). The antimicrobial potential was evaluated by measuring the diameter of the microbial inhibition zones (mm). The samples were tested in triplicate, and the arithmetic mean and standard error were calculated for the obtained values.

##### Antimicrobial Susceptibility Testing by Time-Kill Assay

This is a quantitative method for the evaluation of antimicrobial efficacy adapted for PVA/agar and PVA/agar- EO.A.dr. hydrogel samples. 1 cm^2^ samples were put in contact with 5 mL of a bacterial suspension (0.5 McFarland standard). At each specified contact time (6, 12, 24, 48 h), 1 mL of the bacterial suspension was removed and distributed in sterile Petri dishes. The melted and cooled to 45 °C culture medium was added on top and constantly homogenized (1 min). The positive control sample (T_0_) was represented by the initial bacterial suspension (1.5 × 10^8^/mL). The negative control (C-)was represented by the PVA/agar hydrogel without *Artemisia dracunculus* EO. After curing, the Petri dishes were incubated at 37 °C for 24 h. The evaluation of antimicrobial efficacy consisted of determining the total number of colony-forming units (CFU/mL) and comparing the values obtained at each fixed time with the value of the positive control. To facilitate the evaluation of the results, the CFU/mL values were converted to log_10_ values and expressed as a percentage, where a reduction of 1 log is known to correspond to a bacterial reduction of 90%, while a reduction of 5 log corresponds to a reduction of 99.999%.

## Figures and Tables

**Table 1:** Mean values (mm) and standard errors (SE) of inhibition zones obtained in the antimicrobial assay of *Artemisia dracunculus* essential oil.

*Bacteria Species*	Mean/Standard Error (mm)	
	Ge (10 µg)	EO *Artemisia dracunculus* (10 µL)
*Staphylococcus aureus* (*G*+)	17.3 ± 0.1	9.8 ± 0.1
*MRSA* (*G*+)	0	13.3 ± 0.2
*Enterococcus faecalis* (*G*+)	9.2 ± 0.2	8.7 ± 0.2
*Listeria monocytogenes* (*G*+)	22.3 ± 0.2	7.4 ± 0.1
*Escherichia coli* (*G*−)	19.9 ± 0.2	9.2 ± 0.1
*Klebsiella pneumoniae* (*G*−)	22.2 ± 0.4	7.2 ± 0.1
*Salmonella enteritidis* (*G*−)	20.5 ± 0.2	7.4 ± 0.1
*Salmonella typhimurium* (*G*−)	20.2 ± 0.2	8.3 ± 0.1
*Aeromonas salmonicida* (*G*−)	19.5 ± 0.2	16.9 ± 0.2

Legend: Ge-Gentamycin (10 µg); EO *Artemisia dracunculus*—*Artemisia dracunculus*; Essential oil (*G*+) = Gram-positive bacterial species; (*G*−) = Gram-negative bacterial species; MRSA—Methicillin-resistant *Staphylococcus aureus*.

**Table 2:** Preparation of the PVA/A and PVA/A loaded with *Artemisia dracunculus* essential oil hydrogels.

Samples Code	PVA (mL)	Agar (mL)	*Artemisia dracunculus* (mL)
PVA/A-80/20-0	4	1	0
PVA/A-80/20-0.15	4	1	0.15
PVA/A-80/20-0.3	4	1	0.3
PVA/A-60/40-0	3	2	0
PVA/A-60/40-0.15	3	2	0.15
PVA/A-60/40-0.3	3	2	0.3

## Data Availability

The data that supports the findings of the current study are listed within the article.

## Supplementary Materials

The following supporting information can be downloaded at: https://www.mdpi.com/article/10.3390/gels10010026/s1. Figure S1: Antimicrobial activity of PVA/A loaded with *Artemisia dracunculus* essential oil hydrogels, evaluated with the time-kill assay method; Table S1: Antimicrobial activity of PVA/A EO *Artemisia dracunculus* hydrogels.
