# Supplementary material for: Antimicrobial Activity of Artemisia dracunculus Oil-Loaded Agarose/Poly(Vinyl Alcohol) Hydrogel for Bio-Applications
Source: Gels. 2023 Dec 28;10(1):26. doi: 10.3390/gels10010026 (PMC10815380; doi:10.3390/gels10010026)
Supplement: Supplementary file 1 [file gels-10-00026-s001.zip › gels-2794500-supplementary.pdf]

Table S1. Antimicrobial activity of PVA/A EO *Artemisia dracunculus* hydrogels

Bacteria species	Lg	C (+)	**Hydrogels PVA/A + EO.*Artemisia dracunculus***							
			80/20-0,15		80/20-0,30		60/40-0,15		60/40-0,3	
		$1,5X10^8$ CFU/ml	24h	48 h	24h	48 h	24h	48 h	24h	48 h
Staphylococcus aureus	Lg_{10}	8.176	3,195	0	3,153	0,16	3,091	2,667	2,97	2,356
	Lg red.		4,981	I	5,0229	8,1760	5,0850	5,5092	5,5092	5,8200
	%Lg red.		99,9988	100	99,9990	99,9999	99,9991	99,9996	99,9996	99,9998
MRSA	Lg_{10}	8.176	0,220	0,884	0,801	0,668	3,046	1,505	2,696	0
	Lg red.		7.956	7.292	7.508	7.375	5.129	6.670	5.478	I
	%Lg red.		99.999	99.999	99.999	99.999	99.999	99.999	99.999	100
Enterococcus faecalis	Lg_{10}	8.176	2,426	2,576	3,46	0	3,426	2,647	3,426	2,162
	Lg red.		4,7501	5,5993	4.7163	I	4.7501	5.5290	4.7501	6.0137
	%Lg red.		99,9982	99,9997	99,9980	100	99,9982	99,9997	99,9982	99,9999
Listeria monocytogenes	Lg_{10}	8,176	0,367	0,1694	0	0	0,220	0	2,029	2,004
	Lg red.		7,8087	8,3455	I	I	7,9559	I	6,1467	6,1717
	%Lg red.		99,9999	99,9999	100	100	99,9999	100	99,9999	99,9999
Escherichia coli	Lg_{10}	8,176	4,186	3,669	4,452	4,135	5,362	3,869	4,02	3,948
	Lg red.		3,9904	4,5070	3,7237	4,0414	2,8143	4,3068	4,1562	4,2283
	%Lg red.		99,9897	99,9968	99,9811	99,9909	99,8466	99,9950	99,9930	99,9940
Klebsiella pneumoniae	Log_{10}	8,176	7,337	6,337	5,154	2,196	7,304	5,132	5,278	3,987
	Log red.		0,8389	2,8118	3,0217	5,9801	0,8721	3,0436	2,8981	4,1893
	%Log red.		85,5111	99,8457	99,9048	99,9998	86,5777	99,9095	99,8735	99,9935
Salmonella enteritidis	Lg_{10}	8,176	7,32	0,5635	0,424	4,923	0,1694	0,4776	1,079	0,4776
	Lg red.		0,8559	7,6126	3,2535	3,2535	8,3455	8,6536	7,0969	8,6536
	%Lg red.		86,0666	99,9999	99,9442	99,9442	99,9999	99,9999	99,9999	99,9999
Salmonella typhimurium	Lg_{10}	8,176	2,524	0	2,642	0	4,014	0,1694	2,97	0,4776
	Lg red.		5,6514	8,6536	5,5342	8,6536	4,1618	8,3455	5,2060	8,6536
	%Lg red.		99,9997	99,9999	99,9997	99,9999	99,9931	99,9999	99,9993	99,9999
Aeromonas salmonicida	Lg_{10}	8,176	0	0	0	0	0	0	0	0
	Lg red.		I	I	I	I	I	I	I	I
	%Lg red.		100	100	100	100	100	100	100	100

CFU - colony-forming unit; I- infinity (100% microbial inhibition) , Lg red- log reduction, %Lg red -reduction percentage

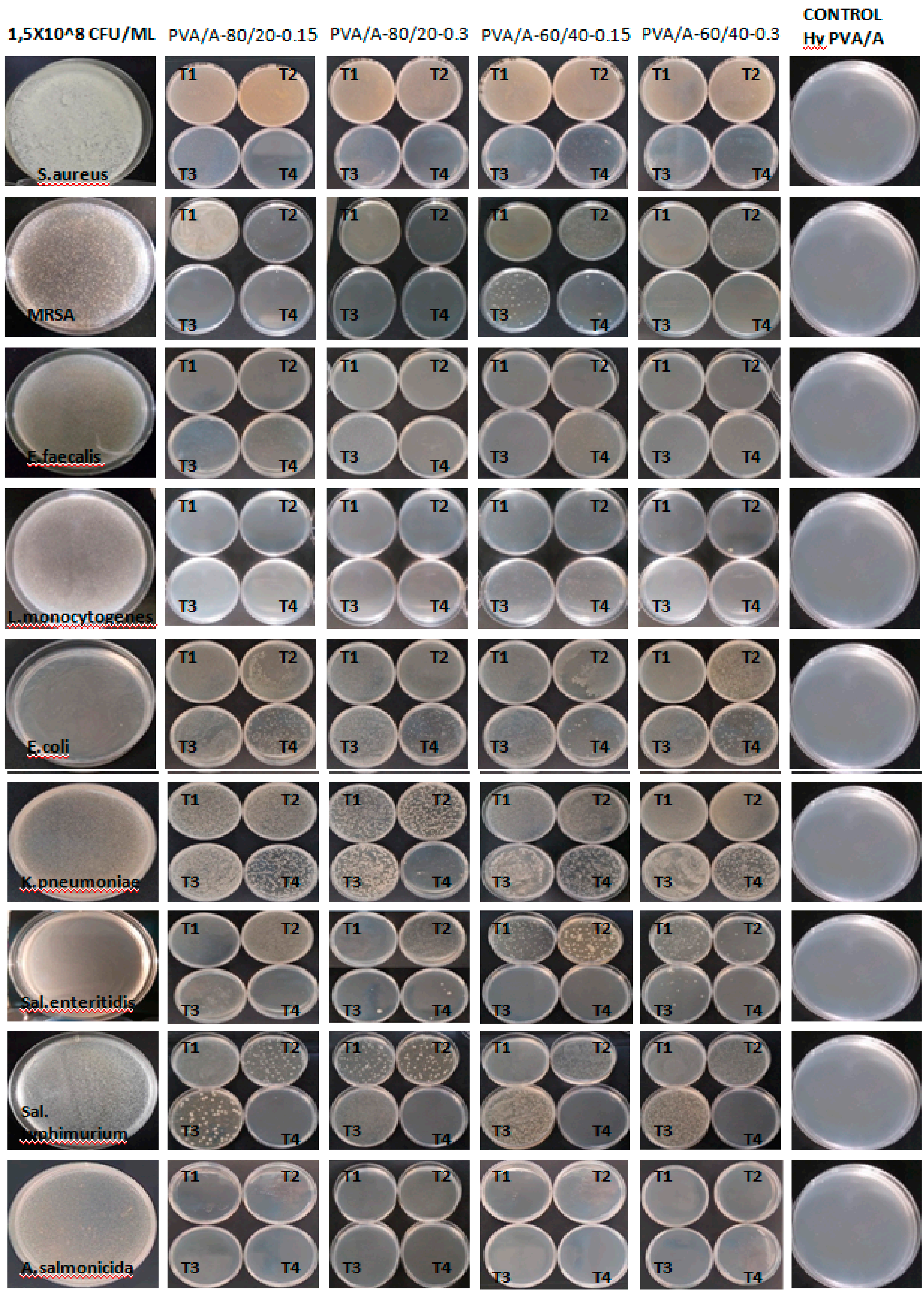

Legend:T1(6 hour), T2 (12 hour); T3 (24 hour), T4 (48 hour); CFU- colony forming unit

Figure S1. Antimicrobial activity of PVA/A loaded with *Artemisia dracunculus* essential oil hydrogels, evaluated with the time-kill assay method
